# Prevalence and factors associated with female sterilization in Rwanda: evidence from the demographic and health survey data (2019–2020)

**DOI:** 10.1186/s12889-023-17334-8

**Published:** 2023-12-07

**Authors:** Samuel Ndayishimye, Gbenga Olorunfemi, Bonfils Nahayo

**Affiliations:** 1 Reproductive Health Programme, Pan African University Life and Earth Sciences Institutes, University of Ibadan, Ibadan, Nigeria; 2 University of Ibadan, Ibadan, Oyo State Nigeria; 3 Division of Epidemiology and Biostatistics, University of Witwatersrand, Johannesburg, South Africa

**Keywords:** Female sterilization, Permanent method, Contraceptives, Rwanda

## Abstract

**Background:**

Female sterilization is a safe and effective surgical procedure of achieving contraception. There is disparity in the prevalence of female sterilization globally, with high income countries having higher rates than low- and middle-income countries. However, current evidence of the uptake of female sterilization in Rwanda is not known. We therefore evaluated the prevalence and factors associated with female sterilization among women of reproductive age in Rwanda.

**Methodology:**

This was a secondary data analysis of 14,634 women of reproductive age (15–49) in Rwanda. The data utilized was from the Rwanda Demographic Health and Survey (RDHS) 2019/2020. The predictors of female sterilization were determined using multivariable binary logistic regression analysis.

**Results:**

We found that the prevalence of female sterilization was 1.1% among women of reproductive age in Rwanda. Women older than 35 years had about 8 times higher chance of being sterilized as compared to younger women (aOR: 7.87, 95% CI: 4.77–12.99). Women living with their partners had higher odds of being sterilized as compared to never married women (aOR: 19.23, 95% CI: 4.57–80.82), while women from minority religion are more likely to be sterilized as compared to those of the catholic religion (aOR: 2.12, 95% CI: 1.03–4.37). Women from rich household had a higher chance to be sterilized as compared to their counterparts from poor households (aOR: 3.13, 95% CI: 1.94–5.03). Women from the Western region were more likely to accept sterilization compared to women from Kigali (aOR: 2.025, 95% CI: 1.17–3.49) and women who had more than 5 children had higher odds when compared to women who had 5 or less children (aOR: 1.49, 95% CI: 1.06–2.10).

**Conclusion:**

The overall prevalence of sterilization among Rwandan women of reproductive age was 1.1%, which was very low as compared to India (29%), China (14.1%) and United States of America (13.7%). The age, marital status, religion, household wealth quintile, region and children ever born were associated with the uptake of female sterilization among Rwandan women. Public awareness campaign on the advantages of female sterilization should be done to improve uptake.

## Introduction

The uptake of modern contraceptive methods has been proven to reduce maternal and perinatal mortality and morbidity and to improve the well-being of the family [1–3]. The prevalence of contraceptive uptake is high in high income countries (HICs) as compared to the low and middle income countries (LMICSs) [4]. Female sterilization was the leading form of contraception globally and constituted about 23.7% (219 million women) of the contraceptive users in 2019. Female sterilization is a surgical procedure of birth control which is highly effective in preventing pregnancy [5–8]. During this procedure, both fallopian tubes are tied, clamped, sealed, or closed to inhibit the ovum from traversing the fallopian tubes in order for it to be fertilized by the spermatozoa [9]. Globally, the prevalence of female sterilization declined from 13.3% in the year 1994 to 11.5% in the year 2019 [4]. However, in the central and southern Asia region, the incidence of female sterilization increased by 4.8% in the last 25 years ago till 2019 [4], and the tubal ligation is the most common form of contraception in the United States of America 13.7% and India 29% [10–12].

Contraceptive uptake in sub-Saharan Africa remains low, with only 28.5% of women of reproductive age using any method of modern contraception [4, 13]. Furthermore, the prevalence of female sterilization in 2019 was low as 1.1% in the sub-Saharan African countries, while injectable contraceptive was the highest method at a rate of 9.6%. Malawi has reported a significantly elevated prevalence of female sterilization in the Sub-Saharan Africa region, with a rate of 18.1%. [14] In the East African countries within the region, the prevalence of female sterilization stands at 2.4% in Kenya and 2% in Uganda. [15].

The existing literature indicates that female sterilization is associated with several factors including the number of living children, HIV/AIDS infection status, participation in counseling campaigns, age, occupation, literacy level, poverty, and culture [15–17]. Due to low prevalence of contraceptive use in the sub-Saharan region, the total fertility rate is higher as compared to other regions of the world. [18, 19] Furthermore, the number of unintended pregnancies as well as the associated complications of unsafe abortions are high [20].

In the last 15 years, contraceptive uptake in Rwanda has increased by about 47% [21]. Recentdata from Rwanda Demographic and Health Survey of the 2019/2020 showed that the prevalence of modern contraceptive uptake was 58%, with the injectable being the most commonly used contraceptive among Rwandan women [22]. The total fertility rate (TFR) declined from 6.8 to 4.2, which is still higher than the desired TFR of 3 children per woman [23]. Fear of side effects, myths, cultural and religious beliefs and lack of knowledge on contraceptives are among factors associated with low uptake of contraceptives in Rwanda [24–27]. Of note, the menstrual irregularity and other side effects linked to hormonal contraceptives usually cause switching or discontinuation of the hormonal injectables and implants [28]. For women who have completed their family size, female sterilization is a simple procedure with the highest efficacy in preventing unwanted pregnancies [29]. However, current evidence on the uptake and determinants of female sterilization is limited in Rwanda. Therefore, this study aimed to determine the prevalence and associated factors of female sterilization in Rwanda.

## Methods

This was a secondary data analysis of 14,634 women of reproductive age (15–49) in Rwanda. Women of reproductive age typically refer to women aged between 15 and 49 years. These individuals are considered to have reached the age of sexual maturity and possess the potential to conceive and bear children. The data utilized was from the RDHS from 2016 to 2020. Rwanda is the second most densely populated country in Africa with the population growth rate of 2.4. About 83% of the population live in rural areas and more than 70% work as subsistence farmers. [30] However, Rwanda is among the fastest growing economy in Africa. [31].

### Data sources

The RDHS is a cross-sectional household survey on the socio-demographic, health and health care status of the Rwandan population. Data was collected using a two-stage cluster sampling method from 2019 to 2020. The initial cluster were randomly selected enumeration areas. Afterwards, households were randomly selected from the enumeration areas. Participants from each selected household were then interviewed.

### Outcome variables

Female sterilization was the outcome variable. Women who had female sterilization were coded as 1 while all other women were coded as 0. Furthermore, the prevalence of female sterilization among all those who were current users of modern contraceptive was also evaluated by obtaining another binary variable in which those who had female sterilization were coded as 1, while women who had other form of modern contraception were coded as 0.

### Explanatory variables

From RDHS data, age of participants was handled as both continuous and binary after being categorized into 2: ≤ 35years and > 35 years. Household wealth quintiles were originally 5 categories: poorest, poor, middle, richer and richest. These wealth quintiles were then re-categorized into 3 categories: poorest and poor was combined to poor, richer and richest was combined to rich categories. “children ever born” was grouped into two categories: ≤5 children and > 5. In terms of employment status, professional jobs and laboured jobs were merged into employed women and non-employment was named as not employed. For religious belief, all religion with less than 5% of the study population were combined and named as other religion. However, region, education level, place of residence and marital status remained unchanged. Selection of the independent variables was based on previous studies related to the uptake of contraceptives and the hypothetical relationship they have with sterilization uptake. [15, 32]

### Data analysis

Data were analyzed using statistical package for social sciences version 25. (SPSS 25, IBM, USA). Descriptive statistics was done. Categorical variables were presented as frequency. The prevalence (with 95% confidence interval) of female sterilization was calculated by dividing the number of women who had female sterilization by the total study population. binary logistic regression was used to assess the association between socio-demographic and reproductive variables and female sterilization. Univariable, bivariate and multivariable binary logistic regression was conducted using stepwise backward elimination technique to obtain crude and adjusted odds ratio of female sterilization. Variables with univariable *P*-value < 0.05 were used to build the multivariable model, and the goodness of fit of the final model was tested by Hosmer-Lemeshow *p*-value > 0.05. Data was survey-set before statistical analysis. Statistical level of significance was set at a 95% CI while two-tailed test of hypothesis was assumed.

## Results

Table 1 shows the socio-demographic and reproductive characteristics of the study participants. Of the 14,634 women of reproductive age group whoparticipated in this study, 70.6% were aged 35 years or younger. Abouthalf of participants were in unions/living with their partners, while more than half of the participants had primary education. Only 4.6% studied more than secondary education. 43.6% were from the rich household wealth quintile. About 10% of women had more than five children. The majority of participants 75.7% were residents in rural area, and 73% of women who participated in this study were employed in professional jobs and laboured jobs.

**Table 1:** Socio-demographic and reproductive characteristics of the study participants

Variables		Frequency N = 14,634	Percentage
Age	≤ 35	10,330	70.6
	> 35	4304	29.4
Marital status	Never in union	6060	41.4
	Currently in union	7290	49.8
	Formerly in union	1284	8.8
Highest educational level	No education	1352	9.2
	Primary	8500	58.1
	Secondary	4110	28.1
	Higher	672	4.6
Religion	Catholic	5506	37.6
	Protestant	6754	46.2
	Adventist	1842	12.6
	Others^*^	532	3.6
Household wealth quintile	Poor	5551	37.9
	Middle	2709	18.5
	Rich	6374	43.6
Type of residence	Urban	3551	24.3
	Rural	11,083	75.7
Region	Kigali	1921	13.1
	South	3482	23.8
	West	3312	22.6
	North	2294	15.7
	East	3625	24.8
Employment	Not working	3955	27.0
	Working	10,679	73.0
Total children ever born	≤ 5	13,169	90
	> 5	1465	10

Other*: Participants from other religions are < 5%

The overall prevalence of female sterilization was 1.1% among women of reproductive age in Rwanda.

A bivariate analysis in Table 2. shows that age, marital status, religion, household wealth quintile, region, parity, highest education level, type of residence and employment status were associated with female sterilization.

**Table 2:** Association between socio-demographic and reproductive characteristics and female sterilization

Variables		OR [95% CI]	Sterilization		*P*-Value
			Yes (%)	No (%)	
Age	≤ 35	1.00	21(0.2)	10,309(99.8)	1.00
	> 35	16.38[10.3–25.9]	139(3.2)	4165(96.8)	< 0.001
Marital status	Never in union	1.00	2(0.0)	6058(100)	1.00
	Currently in union	62.72[15.5-253.4]	148(2.0)	7142(98.0)	< 0.001
	Formerly in union	23.7[5.2-108.6]	10(0.8)	1274(99.2)	< 0.001
Highest educational level	No education	1.00	19(1.4)	1333(98.6)	1.00
	Primary	0.877[0.533–1.44]	105(1.2)	8395(98.8)	0.603
	Secondary	0.395[0.21–0.73]	23(0.6)	4087(99.4)	0.003
	Higher	1.384[0.68–2.82]	13(1.9)	659(98.1)	0.371
Religion	Catholic	1.00	44(0.8)	5462(99.2)	1.00
	Protestant	1.526[1.05–2.20]	82(1.2)	6672(98.8)	0.024
	Adventist	1.639[0.99–2.70]	24(1.3)	1818(98.7)	0.053
	Others^*^	2.378[1.19–4.75]	10(1.9)	522(98.1)	0.014
Household wealth quintile	Poor	1.00	27(0.5)	5524(99.5)	1.00
	Middle	2.368[1.41–3.97]	31(1.1)	2678(98.9)	0.001
	Rich	3.327[2.17–5.09]	102(1.6)	6272(98.4)	< 0.001
Type of residence	Urban	1.00	54(1.5)	3497(98.5)	1.00
	Rural	0.625[0.45–0.87]	106(1)	10,977(99)	0.005
Region	Kigali	1.00	24(1.2)	1897(98.8)	1.00
	South	0.687[0.40–1.18]	30(0.9)	3452(99.1)	0.173
	West	1.310[0.80–2.12]	54(1.6)	3258(98.4)	0.274
	North	0.485[0.25–0.94]	14(0.6)	2280(99.4)	0.032
	East	0.837[0.50–1.40]	38(1)	3587(99)	0.499
Employment	Not working	1.00	29(0.7)	3926(99.3)	1.00
	Working	1.86[1.12–2.51]	131(1.2)	10,548(98.8)	0.012
Total children ever born	≤ 5	1.00	107(0.8)	13,062(99.2)	1.00
	> 5	4.582[3.28–6.40]	53(3.6)	1412(96.4)	< 0.001

OR: odd ratio , other*: Participants from other religions are < 5%

From Table 3, women older than 35 years were about eight times more likely to use permanent contraceptive method as compared to women aged 35 years and younger (aOR: 7.87, 95% CI: 4.77–12.99). Women who were living with their partners and those who were formerly married had 19 and 7 times higher likelihood of uptake of female sterilization compared to women who were never in union (aOR: 19.23, 95% CI: 4.57–80.82) (aOR: 7.112, 95% CI: 1.48–33.99). Odds of female sterilization uptake were twice in minor religious believers compared to the catholic which is more prevalent churches (aOR: 2.12, 95% CI: 1.03–4.37). Women from the rich household quintile were three timesas likely to utilize permanent contraceptive methods as compared to their counterparts from the poor household wealth quintile (aOR: 3.13, 95% CI: 1.94–5.03). Region of residence was associated with female sterilization, as women from Western Provincehad a higher likelihood of undergoing sterilization as compared to women from the Kigali City (aOR: 2.025, 95% CI: 1.17–3.49). Women who had more than 5 children had a 49% higher chance for sterilization compared to their counterparts who had equal to or fewer than 5 children (aOR: 1.49, 95% CI: 1.06–2.10). However, the type of residence, employment status and educational status were not associated with female sterilization.

**Table 3:** Multivariate logistic regression of the factors associated with female sterilization in Rwanda

Variables		AOR	*P*-Value	95% CI
Age	≤ 35	1	Ref	Ref
	> 35	7.87	**< 0.001**	4.77–12.99
Marital status	Never in union	1		
	Currently in union	19.225	**< 0.001**	4.57–80.82
	Formerly in union	7.112	**0.014**	1.48–33.99
Highest educational level	No education	1		
	Primary	1.132	0.637	0.67–1.90
	Secondary	1.130	0.727	0.57–2.25
	Higher	1.417	0.397	0.63–3.18
Religion	Catholic	1	Ref	Ref
	Protestant	1.44	0.59	0.98–2.11
	Adventist	1.47	0.136	0.88–2.46
	Others^*^	2.125	**0.040**	1.03–4.37
Household wealth quintile	Poor	1		
	Middle	2.18	**0.004**	1.28–3.70
	Rich	3.128	**< 0.001**	1.94–5.03
Type of residence	Urban	1	Ref	Ref
	Rural	0.681	0.064	0.45–1.02
Region	Kigali	1	Ref	Ref
	South	1.14	0.659	0.62–2.08
	West	2.025	**0.011**	1.17–3.49
	North	0.764	0.456	0.376–1.551
	East	1.25	0.451	0.701–2.228
Employment	Not working	1	Ref	Ref
	Working	0.832	0.396	0.545–1.27
Total children ever born	≤ 5	1		
	> 5	1.493	**0.022**	1.06–2.10

AOR: Adjusted odd ratio, other*: participants from other religions are < 5%

## Discussion

In this study we assessed the prevalence and factors associated with female sterilization as one of contraceptive methods. We found that the prevalence was very low, with only 1.1% of women of reproductive age in Rwanda having undergone female sterilization. The prevalence of female sterilization remained low in sub-Saharan African countries and some of the reasons included a lack of information, fear of surgical procedures, myths, misconceptions about tubectomy, irreversibility of the procedure and the fear of perceived side effects [32, 33]. Women above 35 years old were more likely to undergo sterilization because the average age for first marriage in Rwanda is 21 years. Thus, by the age of 35 years, many women of reproductive age already had the desired number of children. It is very common that women younger than 35 years use reversible contraceptive method rather than permanent method. [12] Results of this study are in line with findings from studies conducted in Uganda and Ethiopia which revealed that women aged between 35 and 49 years respectively had thirty-six and three times higher odds of female sterilization as compared to younger women [15, 34]. However, this contradicts the report of a study conducted in India, where sterilization uptake was higher in women younger than 30 years old [8]. Women whose belief systems were traditional, non-religious, and minority religions had higher odds of undergoing sterilization compared to women from the Catholic religion. This is attributed to the conservative beliefs influenced by earlier statements from the doctrine of the church against the use of contraception in general. These findings align with a study conducted in Nigeria, which also reported that tubal ligation was opposed by many religious beliefs [35]. The odds of female sterilization were higher among women from rich households compared to their counterparts from poorer households. This can be explained by the wealth flow theory of fertility, where women from poorer households may desire more children in the hope of receiving support from them. [36]. This finding was in line with a study conducted in Zambia, which revealed that women from richer households were more likely to use long acting and non-reversible contraception method compared to women from poor households wealth quintiles [37]. However, it contradicts studies conducted in Uganda and India, where women from poorer families had higher odds of sterilization compared to women from rich household wealth quintiles. [8, 15]Women from the western region were twice as likely to use sterilization as a contraceptive method compared to women from Kigali city. This higher prevalence of non-reversible contraception uptake in the western region can be attributed to the efforts of the government and stakeholders in promoting permanent contraceptive methods, as this region had higher total fertility rate compared to other regions. [38] We found that women who had more than five children had 49% higher odds of opting for permanent contraceptive methods. The choice between reversible and non-reversible methods often depends on the desired number of children. This finding is consistent with a study conducted in Uganda, which reported that women who had not yet reached their desired fertility used reversible contraception methods. [15] It is very common that individuals who have reached their desired fertility use non-reversible methods of contraception. However, in some societies, such as rural areas in Uganda, families may desire more children for different reasons, such as assistance for non-modernized farming. [15].

### Limitation of the study

The findings of this study were based on secondary data analysis, which has limitations in determining all possible determinants of female sterilization. To gain a more comprehensive understanding of the factors associated with permanent methods of contraception, a mixed-method study design for both male and female sterilization would be beneficial.

## Conclusion

The prevalence of female sterilization among women of reproductive age in Rwanda was relatively low at 1.1%. The factors associated with the low prevalence of female sterilization were age, marital status, religion, household wealth quintile, region and number of children ever born. Despite its irreversibility, sterilization is safe and effective method of contraception. Government and stakeholders should enhance mobilization about thesterilization method. Medical practitioners, nurses and other healthcare providers have a sole responsibility to clear myths and misconceptions about sterilization. To effectively reach the national fertility goals and promote the economic empowerment of women, sterilization campaigns should target women of all age groups, enabling them to make informed decisions in a timely manner regarding their desired fertility.

## Data Availability

Data are available upon request from the Demographic and Health Surveys portal: https://dhsprogram.com/data/available-datasets.cfm.

## Abbreviations

ILAPM: Injectables, Long-Acting and Permanent Methods
RDHS: Rwanda Demographic Health and Survey
TFR: Total fertility rate
CI: Confidential Interval
OR: odd ratio
aOR: adjusted odd ratio
